# The Efficacy of Using Peer Mentors to Improve Maternal and Infant Health Outcomes in Hispanic Families: Findings from a Randomized Clinical Trial

**DOI:** 10.1007/s10995-018-2532-z

**Published:** 2018-05-31

**Authors:** Melanie Lutenbacher, Tonya Elkins, Mary S. Dietrich, Anais Riggs

**Affiliations:** 1 Schools of Nursing and Medicine (Pediatrics), Vanderbilt University, Nashville, TN USA; 2 Maternal Infant Health Outreach Worker Program, Vanderbilt University, Nashville, TN USA; 3 Schools of Medicine (Biostatistics, Psychiatry, VICC) and Nursing, Vanderbilt University, Nashville, TN USA; 4 Catholic Charities of Tennessee, Inc., Nashville, TN USA

**Keywords:** Hispanic, Home visit, Prenatal, Depressive symptoms, Peer mentors, Safe sleep

## Abstract

*Introduction* The Maternal Infant Health Outreach Worker (MIHOW) program is a home visiting program, utilizing peer mentors to improve maternal/child health outcomes in underserved communities. Findings are presented from a randomized clinical trial (RCT) testing the efficacy of the MIHOW model in a sample of Hispanic women in Tennessee. We hypothesized maternal and infant outcomes would be better in women assigned to MIHOW than women assigned to the minimal education intervention (MEI) group (receipt of educational materials). *Methods* Women entered the study during pregnancy (< 26 weeks gestation) and were followed through 6 months postpartum. A total of 188 women were enrolled and randomly assigned (MEI = 94; MIHOW = 94), with 178 women completing the study (MEI = 87; MIHOW = 91). *Results* Positive and statistically significant (p < 0.01) effects of MIHOW were observed on breastfeeding self-efficacy and exclusivity, levels of depressive symptoms and parenting stress, safe sleep practices, and infant stimulation in the home. No statistically significant differences were noted in number of prenatal visits. *Discussion* Results expand limited empiric evidence and provide strong support of the effectiveness of MIHOW on improving health outcomes in this sample of Hispanic mothers and their infants. MIHOW is a viable option for providing culturally sensitive services to immigrant and underserved families.

## Significance

Peer mentors and home visiting may be an effective strategy for immigrant women and their infants but little rigorous evidence exists and findings are inconclusive. Findings from this RCT suggest that a series of home visits by peer mentors beginning during pregnancy until 6 months postpartum is an effective intervention in reducing depressive symptoms and parenting stress, and improving social and emotional support in Hispanic women. Women who received MIHOW also exclusively breastfed their infants longer, had higher rates of exclusive breastfeeding, placed their babies on their backs more often and co-slept with their infants less frequently than women in a minimal education intervention group.

## Introduction

The Maternal Infant Health Outreach Worker (MIHOW) program began in 1982 as a program to address the lack of healthcare in low income, isolated communities in Appalachia. The program goals were to improve maternal health and child development, combat isolation and increase access to health care. The key component of this model is the outreach worker or peer mentor. These are women recruited from the target community of the same race, culture and language, who have strong problem-solving and communication skills and familiarity with resources (Elkins et al. 2013). The peer mentors receive intensive training to provide health education, social support and linkage to community resources. Since its beginning over 30 years ago, MIHOW has served an estimated 15,000 families in the Southeastern United States (US) (Elkins et al. 2013).

Since the 1950s, Hispanic promotoras, similar to MIHOW peer mentors, have worked to reduce and eliminate health disparities (Andrews et al. 2004; Koniak-Griffin et al. 2015; O’Brien et al. 2010; Tran et al. 2014). Promotor as have targeted a variety of health problems, providing health education, helping families navigate systems, making referrals, and sometimes even directly delivering medical services, but rigorous evaluation of efforts is limited (O’Brien et al. 2010).

Currently, MIHOW is considered a promising approach as it meets the criteria defined by the federal Social Security Act, Title V, § 511 [42 U.S.C. § 711] (c). Specifically, MIHOW uses a research-based curriculum, was developed by an institution of higher education, and the approach works to achieve the benchmark areas and outcomes specified in the act. Further, the MIHOW program has demonstrated some effectiveness based upon program evaluation (Clinton 1992; Elkins and Clinton 2009). However, empiric studies are needed to determine the program’s impact and to be qualified as an evidence-based program (HRSA 2016). For more information on MIHOW, visit http://www.mihow.org.

This report presents findings from a randomized clinical trial (RCT) that tested the home visiting model by peer mentors in improving selected maternal and infant health outcomes in a sample of pregnant Hispanic women living in a large city in Tennessee. Our global hypothesis was that women (and their infants) randomly assigned to receive the MIHOW Program would have better health outcomes than those women who were assigned to a minimal education intervention group (MEI—received printed educational materials only). Specifically, the team hypothesized that the mothers receiving the MIHOW intervention would be more likely than the comparison group to: (1) breastfeed longer; (2) delay feeding their infants solids; (3) put babies to sleep on their backs; (4) attend more prenatal care visits; (5) report lower levels of parental stress; (6) report fewer maternal depressive symptoms; (7) receive more referrals; (8) report higher levels of parental support; and, (9) read to their babies more often.

Hispanic families, the largest ethnic minority in the US (U.S. Census Bureau & Population Division 2013), do not have the same access to health care as non-Hispanic whites (Kirby and Kaneda 2013). Lack of access can be influenced by immigration status, socioeconomic status, low education, limited English proficiency, or other social determinants of health (Velasco-Mondragon et al. 2016).

The majority (68.3%) of Hispanic pregnant women in the US start prenatal care in the first trimester (U.S. Department of Health and Human Services 2013). Early prenatal care can identify and rectify maternal and infant risks. Studies of prenatal care usage among Hispanic women in California, New York, and Florida suggest that they are less likely to adequately use prenatal services than US born citizens (Fuentes-Afflick et al. 2006) but because the majority initiate prenatal care early, pregnancy may be an excellent time to engage Hispanic families in a structured health promoting program such as MIHOW. However, few studies have examined the effectiveness of home visitation programs in Hispanic families (O’Brien et al. 2010).

Hispanic women experience health disparities related to depression identification and treatment (Baker-Ericzen et al. 2012). Rates of depressive symptoms have been reported between 20 and 38% in samples of Hispanic/Latino women in Florida, California, and Massachusetts (Gress-Smith et al. 2012; Wassertheil-Smoller et al. 2014). Left untreated, depression can have significant consequences for the woman and impact her ability to effectively parent her children. Empiric evaluation of the impact of a home visitation program on the mental health of mothers is lacking and is rare in Hispanic populations (Gomby 2005).

Breastfeeding benefits are widely known (Flores et al. 2016; Vaughn et al. 2010; Victora et al. 2016). While Hispanic mothers in the US tend to initiate breastfeeding at high rates, they also supplement with formula at high rates despite recommendations to exclusively breastfeed (Jones et al. 2015). Women who receive prenatal education and home based postpartum support are more likely to initiate breastfeeding and continue to breastfeed for 6 months (Gill et al. 2007). In a study of predominantly low-income Dominican women, higher breastfeeding self-efficacy scores were associated with more breastfeeding and exclusive breastfeeding (Glassman et al. 2014). Strategies, such as MIHOW, that encourage Hispanic mothers’ choice to breastfeed exclusively and through 6 months need to be evaluated.

While more than 90% of Hispanic children in the US are US citizens, Hispanic children disproportionately live in poverty, suffer from health problems such as overweight/obesity, and enter school inadequately prepared (Murphey et al. 2014). Hispanic infants are less likely than White non-Hispanic children to be read to by their parents on a daily basis (Federal interagency forum on child and family statistics 2017). Despite many barriers to healthy outcomes, most Hispanic mothers report compliance with American Academy of Pediatrics (AAP) recommended infant safe sleep practices supporting back sleeping and discouraging co-sleeping practices (Provini et al. 2017).

## Methods

This single site, randomized clinical trial was approved by the Institutional Review Board (IRB) of Vanderbilt University Medical Center in the Southeastern US.

### Participants and Sample Size Justification

To be enrolled in the study, women had to: be eligible to receive MIHOW services; self-identify as Hispanic; provide written confirmation of pregnancy ≤ 26 weeks gestation; reside within 30 miles of the study offices; and be willing to be randomized into one of two study groups. Women were excluded from the study if they had previously received MIHOW services; had a severe mental or physical disability; or were under 18 years of age.

**Fig. 1:**
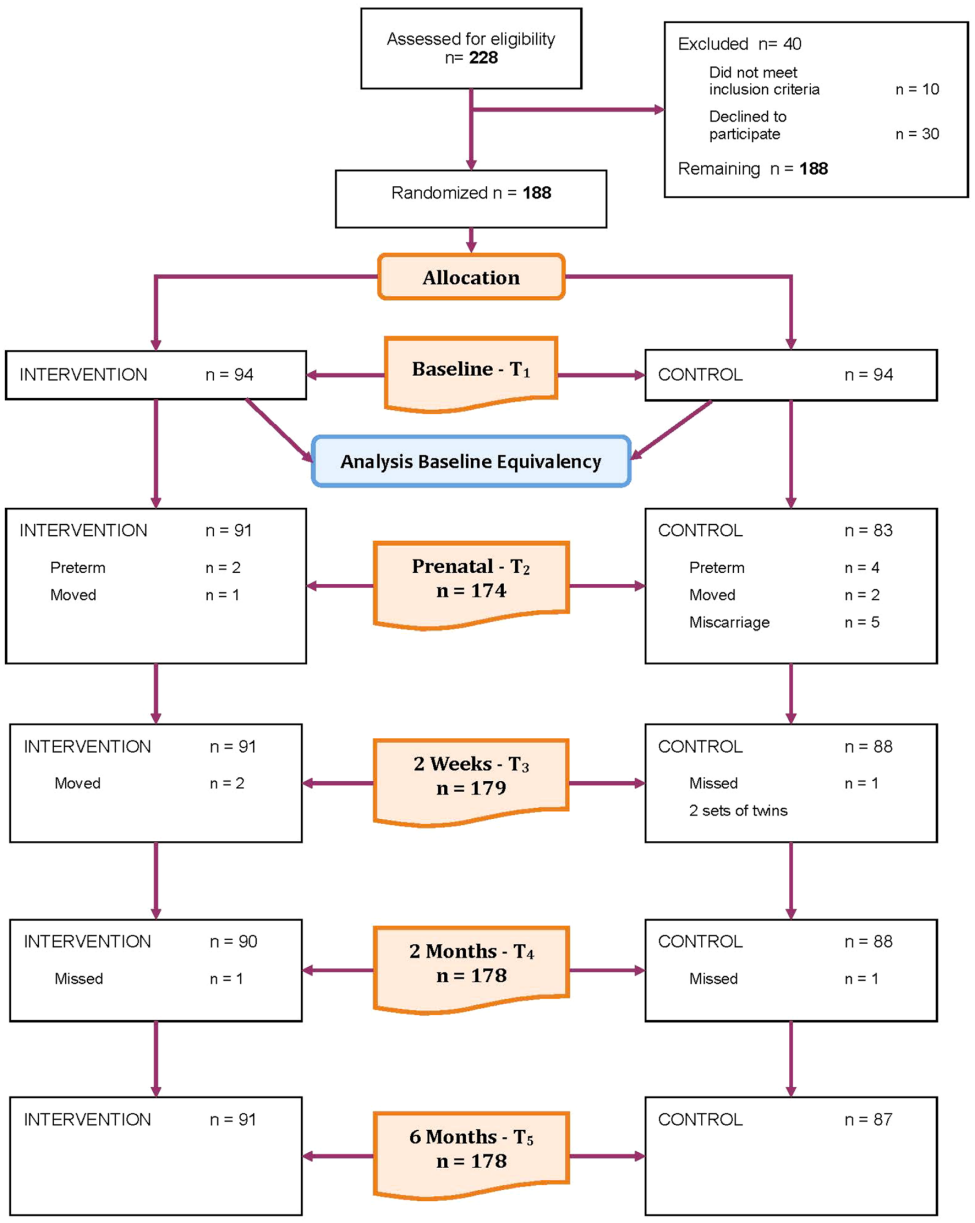
CONSORT flow diagram of the progress through the phases of the randomized trial

Study participants were 188 pregnant Hispanic women living in a large metropolitan area in (blinded) Tennessee. The original sample size for this study was justified by both the prior experience of the research team and the MIHOW program and a conservative estimate of the number of participants that could complete the protocol within the study period (based on typical MIHOW participation numbers). That original number was 150 (75 per study group). A statistical powering analysis revealed that groups of that size were sufficient to detect common Cohen’s d effect size of 0.46 (80% power, 2-sided alpha = 0.05). All effects would be translated into this common index. A Cohen’s d of that magnitude was deemed clinically meaningful and therefore further justified our sample size goal. Recruitment and participant interest as the study progressed was such that a decision was made by the research team to continue enrollment as long as the protocol could allow. Ultimately, a total of 188 women were enrolled. Of those women, 178 completed the study. For study consort flow diagram, see Fig. 1.

### Procedure

Upon receiving IRB approval for the study protocol, recruitment began with a variety of recruitment strategies including distributing flyers at locations with a high volume of Hispanic customers (e.g., clinics, markets, apartment complexes, churches) and by word of mouth. All women interested in participating in the study were screened by trained study staff to determine eligibility. All eligible women interested in participating in the study completed a written informed consent. Group assignments were generated by the study statistician via a computer-generated, permuted block program. Participants received their group assignment after the enrollment interview was completed. All data were collected by trained study staff who were women hired specifically for this project from the local communities who were both linguistically (i.e., native Spanish speakers and fluent in English) and culturally competent. Data collectors were ‘blind’ to group assignment. All study staff completed extensive training related to the conduct of a randomized clinical trial and to study protocols. Data collectors used an interview guide at 5 points: enrollment (≤ 26 weeks pregnant), approximately 35 weeks pregnant, and 2 weeks, 2 months and 6 months postpartum. Interview guides were available in Spanish and English.

Each data collection interview took approximately 1 h. With the exception of the *HOME* measure, data collectors read all questions and items aloud to women and used a paper and pencil format to complete the interview guides. The *HOME* was completed via observation of the data collector at each of the postpartum data collection points. All participants received a $25 merchandise card at the end of each interview. Staff entered data into a REDCap database located on a secure password protected server at the associated university. Monthly data fidelity checks of a random selection of data forms were conducted by the project coordinator. Data were collected between July 2014 and September 2016.

### Comparison Condition

The comparison condition (minimal education intervention: MEI) consisted of distribution of printed educational materials about maternal and infant health and development at the end of each data collection interview to all study participants (i.e., women assigned to both study groups) in order to maintain the blind status of data collectors. Materials were available in Spanish or English.

### Intervention Condition

The intervention condition included the core elements of the MIHOW model. No adaptations were made to MIHOW content, level of intensity, or home visitor training requirements. MIHOW interventionists were recruited from the local Hispanic community who completed 40 h of training to the MIHOW curriculum. The MIHOW model stresses recognizing family strengths and utilizing those to address their own family needs (Elkins et al. 2013); relationships begin in pregnancy and consist of monthly home visits and periodic group gatherings. MIHOW protocols include listening to maternal concerns, educating about objectives relevant to the woman’s stage of pregnancy or the age of the child, such as healthy eating, developmental milestones, attachment, and breastfeeding, and helping provide links to needed medical and social services. Home visits typically last approximately 1 h. Due to the study’s limited funding, the duration of MIHOW services was limited to pregnancy through 6 months of age rather than the typical duration to the child’s third birthday.

### Measures

Primary outcomes were assessed with standardized measures and established questions from national sources (e.g., 2011/12 National Survey of Children’s Health (NSCH), http://childhealthdata.org/learn/NSCH/topics_questions; Pregnancy Risk Assessment Monitoring System (PRAMS) Phase 6, https://www.cdc.gov/prams/questionnaire.htm). See Table 1 for study variables, measures and the time points for data collection. Standardized measures are briefly described below.

**Table 1:** Study variables, measures, and data collection time points by domain

Variable	Measure/question(s)
Child health domain	
Breastfeeding initiation	• Did you ever breastfeed or pump breast milk to feed your new baby after delivery, even for a short period of time? (Source: PRAMS)
	Time point: ~2 weeks pp
Breastfeeding duration and exclusivity	• Are you currently breastfeeding or feeding pumped milk to your new baby? (Source: PRAMS)
	• How old was your baby when she/he completely stopped breastfeeding or being fed breast milk? (Source: NSCH)
	• How old was your baby when she/he was first fed formula?
	• Over the last 24 h, how many times did you breastfeed your baby?
	• Over the last 24 h, how many times did the baby receive formula? (Source: NSCH)
	Time point: ~2 months pp, ~ 6 months pp
Breastfeeding self-efficacy	• Breastfeeding self-efficacy scale (BSES-SF) (Dennis 2003)
	Time point: enrollment, ~ 2 weeks pp, ~ 6 months pp
Introduction of solid foods	• How old was your baby when she/he was first fed anything other than breast milk of formula? (Source: NSCH)
	• How old was your new baby the first time he or she ate food, such as baby cereal, baby food, or any other food? (Source: PRAMS)
	• How often have you added cereal to your baby’s bottle in the past 2 weeks? (Source: IFPS II)
	Time point: ~2 weeks pp, ~ 2 months pp, ~ 6 months pp
Infant safe sleep	• How do you most often lay your baby down to sleep now?
	• How often does your new baby sleep in the same bed with you or anyone else? (Source: PRAMS)
	Time point: ~2 weeks pp, ~ 2 months pp, ~ 6 months pp
Prenatal care visits	• How many weeks or months pregnant were you when you had your first visit for prenatal care? (Source: PRAMS)
	• How many prenatal visits did you have during the entire pregnancy? (ask at ~ 2 weeks only)
	Time point: enrollment, ~ 35 weeks, ~ 2 weeks pp
Maternal health domain	
Parenting stress and support	• Parenting stress index 4—short form (Abidin 2012)
	How often do you get the social and emotional support you need?
	Time point: enrollment, ~ 2 weeks pp, ~ 2 months pp, ~ 6 months pp
Maternal depression	• Edinburgh postpartum depression scale (Cox et al. 1987)
	Time point: enrollment, ~ 35 weeks, ~ 2 weeks pp, ~ 2 months pp, ~ 6 months pp
Linkages and referrals domain	
Follow through with referrals	• Since you started the study, has anyone talked to you about services or resources in your community you may qualify for (such as WIC, a food bank, legal or immigration services, or a children’s group)?
	• If yes, which services/resources did they talk to you about?
	• Have you called or visited any of the places they told you about?
	• Have you received any new services as a result of the referral?
	• If yes, list the services
	Time point: ~35 weeks, ~ 6 months pp
Positive parenting domain	
Parenting practices	• HOME inventory (Caldwell and Bradley 1984)
	Time Point: ~2 weeks pp, ~ 2 months pp, ~ 6 months pp
Reading and singing	• During the past week, how many days did you or other family members tell stories or sing songs to your baby?
	• During the past week, how many days did you or other family members read to your baby? (Source: NSCH)
	Time point: ~2 months pp, ~ 6 months pp

#### Breastfeeding Self-Efficacy Scale-Short Form (BSES-SF) (Dennis 2003)

The 14-item measure assesses a mother’s confidence in her ability to breastfeed her new infant and has been evaluated among women from diverse cultures with adequate reliability and validity (Dennis 2003; Vaughn et al. 2010). Cronbach’s alpha of the scores in the current study ranged from 0.93 to 0.95.

#### Parenting Stress Index-Short Form (PSI) (Abidin 2012)

PSI was used to assess the level of stress in the parent–child system. This 36-item scale includes three domains: parental distress (PD), parent–child dysfunctional interaction (P–CDI), and difficult child (DC) that when combined form a total stress scale. Available in Spanish, previous reports indicate strong reliability coefficients (Barroso et al. 2016). Reliability coefficients of the scores in the current study ranged from 0.88 to 0.89.

#### Edinburgh Postnatal Depression Scale (EPDS) (Cox et al. 1987)

This widely used 10-item scale utilizes a 4-point response set to measure level of depressive symptoms with a possible range of scores from 0 to 30. Most research on the EPDS indicates a cut-off of 13 to indicate high depressive symptoms (Cox et al. 1996, 1987). Cronbach’s alphas of the scores in the current study ranged from 0.87 to 0.88.

#### Home Observation for Measurement of the Environment–Infant-Toddler (HOME-IT) (Caldwell, 1984)

The 45-item observational HOME-IT Inventory assesses the quality and quantity of stimulation and support available to a child (birth to age three) in the home environment. The measure contains 6 subscales that assess specific parenting behaviors that support child learning and development including: responsivity; acceptance; organization; learning materials; involvement; and variety in environment (Caldwell and Bradley 1984). Adaptations to some items on the scale were made for infants < 6 months.

### Statistical Analyses

SPSS software was used to summarize study data and test hypotheses. All analyses were done using intention-to-treat principles. Descriptive statistics and plots were used to summarize and initially inspect the distributions of demographic and study measures at each time of assessment. For those measures assessed more than once over the course of the study, values of change in those measures were also generated and summarized. Frequency distributions summarized nominal and ordinal distributions; means and standard deviations summarized normal continuous distributions, median and inter-quartile range (IQR) skewed distributions. Information from those descriptive and graphical evaluations was used to determine the most appropriate test distribution to specify within the mixed-effects generalized linear models used for testing study hypotheses (e.g., normal, log with a Tweedie, etc.). Within these models, the interaction effect of study group and time of assessment (controlling for baseline values) provided the critical test of differences between the study groups in the amount of change in an outcome measures. Effect sizes were generated for all of the comparisons using Cohen’s d statistic. Statistical significance tests maintained maximum type I error rates (alpha values) of < 0.05.

## Results

### Sample Characteristics

The average maternal age at enrollment was 29.6 years (SD = 6.5). Most women reported a Mexican heritage (66.9%), less than a high school education (80.6%), never marrying (56.7%), and annual incomes less than $15,000 (96.6%). Both study groups had similar sociodemographic and scores for standardized measures used to assess outcomes. See Table 2 for detailed sample characteristics.

**Table 2:** Demographic characteristics at enrollment (N = 178)

	Overall (N = 178)	MEI (N = 87)	MIHOW (N = 91)	*p* value
	Mean (SD)			
Age (years)	29.6 (6.5)	28.7 (6.3)	30.4 (6.6)	0.093
Nation of origin	N (%)			0.281
Costa Rica	1 (0.6)	0 (0.0)	1 (1.1)	
El Salvador	17 (9.6)	8 (9.2)	9 (9.9)	
Guatemala	12 (6.7)	3 (3.4)	9 (9.9)	
Honduras	28 (15.7)	17 (19.5)	11 (12.1)	
Mexico	119 (66.9)	59 (67.8)	60 (65.9)	
Peru	1 (0.6)	0 (0.0)	1 (1.1)	
Employment status*				< 0.001
Full-time	17 (9.6)^a^	16 (18.4)^b^	1 (1.1)	
Part-time	28 (15.7)	16 (18.4)	12 (13.2)	
Unemployed/looking	2 (1.1)	1 (1.1)	1 (1.1)	
Unemployed/not looking	131 (73.6)^a^	54 (62.1)^b^	77 (84.6)	
Marital status				0.066
Married	70 (39.3)	40 (46.0)	30 (33.0)	
Separated, divorced, widowed	7 (3.9)	0 (0.0)	7 (5.7)	
Never married	101 (56.7)	47 (54.0)	54 (59.3)	
Highest grade completed	*N* = *176*		*N* = *89*	0.334
8th grade or less	71 (40.3)	36 (41.4)	35 (39.3)	
9th–12th grade, no diploma	71 (40.3)	31 (35.6)	40 (44.9)	
High school diploma/GED	34 (19.3)	20 (23.0)	14 (15.7)	
Family income				0.599
< $10,000	122 (68.5)	57 (65.5)	65 (71.4)	
$10,001–$15,000	50 (28.1)	27 (31.0)	23 (25.3)	
$15,001–$40,000	6 (3.4)	3 (3.4)	3 (3.3)	
	Median [IQR] (min, max)			
Months in U.S.	108.0 [36–156] (1, 408)	108.0 [48–144] (2, 318)	120.0 [36–156] (1, 408)	0.504
Months in (blinded)	89.5 [24–132] (1, 288)	84.0 [24–124] (1, 264)	96.0 [24–132] (1, 288)	0.526
Number of children in home	2.0 [1–3] (0, 5)	2.0 [1–3] (0, 5)	2.0 [1–3] (0, 5)	0.812
Number of adults and children in home	4.0 [3–6] (1, 9)	4.0 [3–6] (2, 9)	4.0 [4–5] (1, 9)	0.751

No respondents received unemployment or worker’s compensation

*Superscripts indicate statistically significant post-hoc pairwise comparisons, Bonferroni-corrected, *p* < 0.05

### Primary Outcomes

#### Outcomes in the Child Health Domain

Summaries of infant feeding practices outcomes at 6 months postpartum are shown in Table 3. The strongest effects of the MIHOW program were observed on the BSES-SF scores and on the rates and duration of breastfeeding exclusivity (Cohen’s d = 0.38–0.76). Approximately 80% (n = 68 of 86, 79.1%) of the women in the MEI group reported never breastfeeding exclusively. That respective percentage was considerably lower in the MIHOW group (n = 50 of 90, 55.6%, d = 0.38, p = 0.011). The difference between the groups in duration was a median 1.4 weeks, with 25% of the MIHOW group exclusively breastfeeding for at least 6 weeks (d = 0.42, p = 0.005). The effects of the MIHOW program on breastfeeding self-efficacy occurred between baseline and the initial postpartum assessment at 2-weeks with the MIHOW group scores being higher at that assessment and maintaining that difference throughout the 6 month postpartum period (see Table 3). A related but secondary outcome was noted to be significant. While the median time to initiation of other liquids was the same for both groups (20 weeks), a higher proportion of the MIHOW group delayed the initiation of other liquids (d = 0.59, p < 0.001). 50% of the MEI women initiated other liquids between weeks 16–20 postpartum; that respective interval was 18–22 weeks for the women in the MIHOW group (see Table 3).

**Table 3:** Summaries of infant feeding practices by study group (N = 178)

	Overall N = 178	MEI N = 87	MIHOW N = 91	*p* value
	N (%)			
	*N* = *177*	*N* = *86*	*N* = *91*	
Ever breastfed	149 (84.2)	71 (82.6)	78 (85.7)	0.565 (*d* = 0.13)
Breastfeeding status	*N* = *175*	*N* = *85*	*N* = *90*	0.762 (*d* = 0.01)
Breastfeeding—6 months PP	87 (49.7)	42 (49.4)	45 (50.0)	
Never breastfed	28 (16.0)	15 (17.6)	13 (14.4)	
Stopped by 2 weeks PP	4 (2.3)	2 (2.4)	2 (2.2)	
Stopped by 2 months PP	22 (12.6)	8 (9.4)	14 (15.6)	
Stopped by 6 months PP	34 (19.4)	18 (21.2)	16 (17.8)	

	Median [IQR] (Min, Max)			
	*N* = *146*	*N* = *70*	*N* = *76*	
Breastfeeding duration in weeks	28.0 [12–28] (1, 28)	28.0 [12–28] (1, 28)	28.0 [12–28] (2, 28)	0.754 (*d* = 0.05)
	N (%)			
Breastfeeding exclusivity	*N* = *176*	*N* = *86*	*N* = *90*	0.011 (*d* = 0.38)
Never	118 (67.0)	68 (79.1)^a^	50 (55.6)^b^	
Stopped by 2 weeks PP	31 (17.6)	10 (11.6)^a^	21 (23.3)^b^	
Stopped by 2 months PP	24 (13.6)	7 (8.1)^a^	17 (18.9)^b^	
Still exclusive 6 months PP	3 (1.7)	1 (1.2)	2 (2.2)	
Exclusive breastfeeding duration in weeks	0.4 [0–4] (0, 28) *175*	0.3 [0–2] (0, 28) *86*	1.4 [0–6] (0, 28) *89*	0.005 (*d* = 0.42)
Time to first other liquid in weeks	20.0 [16–22] (2, 24) *161*	20.0 [16–20] (2, 24) *79*	20.0 [18–22] (4, 24) *82*	< 0.001 (*d* = 0.59)
Time to first food in weeks	20.0 [20–22] (1, 24) *163*	20.0 [20–22] (1, 24) *80*	21.0 [20–22] (12, 24) *83*	0.201 (*d* = 0.19)

	Median [IQR] *N*			
BSES scores				< 0.001^a^ (*d* = 0.76)
Baseline	54.0 [50–60] 127	53.0 [50–60] *66*	54.0 [50–61] *61*	
2 weeks PP	56.0 [51–65] *145*	52.0 [48–56] *69*	61.0 [56–66] *76*	
2 months PP	56.0 [51–63] *121*	51.0 [46–56] *60*	62.0 [56–65] *61*	
6 months PP	57.5 [52–64] *88*	53.0 [48–55] *42*	64.0 [58–66] *46*	

^a^Interaction effect of study group and time of assessment, no statistically significant difference at baseline, MIHOW > MEI 2 weeks, 2 and 6 months

Safe sleeping practices reported by the women in each of the study groups are summarized in Table 4. The women in the MIHOW group were much more likely to report positioning the infant on the back than did the women in the MEI group (~ 98 vs. 66–75%, d = 0.63, p < 0.001). A related but secondary outcome was significant. The MIHOW group of women reported ‘Never’ practicing co-sleeping with the infant more than did the MEI group (81–86% vs. 28–33%, d = 1.17, p < 0.001, see Table 4).

**Table 4:** Summaries of sleeping practices by study group (N = 178)

	Overall N = 178	MEI N = 87	MIHOW N = 91	*p* value
	N (%)			
Sleep position				< 0.001^a^ (*d* = 0.63)
2 weeks PP	*N* = *177*	*N* = *86*	*N* = *91*	
On back	147 (83.1)	57 (66.3)	90 (98.9)	
On side	30 (16.9)	29 (33.7)	1 (1.1)	
On stomach	0	0	0	
2 months PP	*N* = *176*	*N* = *86*	*N* = *90*	
On back	145 (82.4)	57 (66.3)	88 (97.8)	
On side	30 (17.0)	28 (32.6)	2 (2.2)	
On stomach	1 (0.6)	1 (1.2)	0 (0.0)	
6 months PP				
On back	154 (86.5)	65 (74.7)	89 (97.8)	
On side	23 (12.9)	21 (24.1)	2 (2.2)	
On stomach	1 (0.6)	1 (1.1)	0 (0.0)	
Co-sleeping				< 0.001^b^ (*d* = 1.17)
2 weeks PP	*N* = *177*	*N* = *86*	*N* = *91*	
Always	21 (11.9)	21 (24.4)	0 (0.0)	
Often	16 (9.0)	13 (15.1)	3 (3.3)	
Sometimes	17 (9.6)	13 (15.1)	4 (4.4)	
Rarely	21 (11.9)	11 (12.8)	10 (11.0)	
Never	102 (57.6)	28 (32.6)	74 (81.3)	
2 months PP	*N* = *176*	*N* = *86*	*N* = *90*	
Always	18 (10.2)	17 (19.8)	1 (1.1)	
Often	12 (6.8)	12 (14.0)	0 (0.0)	
Sometimes	20 (11.4)	18 (20.9)	2 (2.2)	
Rarely	24 (13.6)	15 (17.4)	9 (10.0)	
Never	102 (58.0)	24 (27.9)	78 (86.7)	
6 Months PP				
Always	17 (9.6)	16 (18.4)	1 (1.1)	
Often	15 (8.4)	13 (14.9)	2 (2.2)	
Sometimes	18 (10.1)	15 (17.2)	3 (3.3)	
Rarely	22 (12.4)	15 (17.2)	7 (7.7)	
Never	106 (59.6)	28 (32.2)	78 (85.7)	

^a^Main effect of study group (on back: MIHOW > MEI, p < 0.001)

^b^Main effect of study group (never: MIHOW > MEI, p < 0.001)

#### Outcomes in the Maternal Health Domain

Ninety-nine percent received prenatal care beginning at approximately 13 weeks gestation with about a total number of nine prenatal visits. No statistically significant differences between the groups (d = 0.04–0.12) were found. Median maternal depressive symptom scores were 7.0 (of possible 30) at baseline with essentially equivalent group variability in those scores (see Table 4). Compared to the MEI group, women in the MIHOW group demonstrated a statistically significant greater decrease in scores between the baseline and prenatal assessments with the values remaining lower throughout the postpartum period (d = 0.57, p < 0.001). Parenting stress and support were only assessed postpartum. Those scores are also summarized in Table 5. As shown, relative to the women in the MEI group, women in the MIHOW group reported lower levels of parenting stress and higher levels of available social and emotional help (d = 0.43 and 0.39 respectively, p < 0.001).

**Table 5:** Summaries of prenatal care, maternal depressive symptoms, stress, and support by study group (N = 178)

	Overall N = 178	MEI N = 87	MIHOW N = 91	*p* value
Prenatal care	N (%)			
	*N* = *172*	*N* = *83*	*N* = *89*	
Receiving prenatal care				0.960 (*d* = 0.04)
No	2 (1.2)	1 (1.2)	1 (1.1)	
Yes	170 (98.8)	82 (98.8)	88 (98.9)	

	Mean (SD) (Min, Max) *N*			
Time to first prenatal visit (weeks)	13.0 (5.0) (3, 26) *170*	12.7 (5.0) (3, 26) *82*	13.3 (4.9) (3, 25) *88*	0.397 (*d* = 0.12)

	Median [IQR] *N*			
EPDS				< 0.001^a^ (*d* = 0.57)
Baseline	7.0 [2–10] *178*	7.0 [3–9] 87	7.0 [2–10] 91	
Prenatal	1.0 [0–6] *172*	4.0 [0–7] *83*	0.0 [0–2] *89*	
2 weeks PP	2.0 [0–5] *177*	5.0 [2–8] *86*	0.0 [0–1] *91*	
2 months PP	0.0 [0–3] *176*	3.0 [0–6] *86*	0.0 [0–0] *90*	
6 months PP	0.0 [0–1] *178*	0.0 [0–4] *87*	0.0 [0–0] *91*	
PSI total stress				< 0.001^b^ (*d* = 0.43)
2 weeks PP	75.0 [72–80] *177*	76.0 [74–81] *86*	74.0 [66–79] *91*	
2 months PP	75.0 [72–80] *176*	76.0 [74–81] *86*	74.0 [68–79] *90*	
6 months PP	75.0 [73–81] *178*	77.0 [75–82] *87*	74.0 [70–79] *91*	

	N (%)			
Social and emotional help				< 0.001^c^ (*d* = 0.39)
2 weeks PP	*N* = *177*	*N* = *86*	*N* = *91*	
Always	146 (82.5)	58 (67.4)	88 (96.7)	
Usually	30 (16.9)	27 (31.4)	3 (3.3)	
Sometimes	1 (0.6)	1 (1.2)	0 (0.0)	
Never	0 (0.0)	0 (0.0)	0 (0.0)	
2 months PP	*N* = *176*	*N* = *86*	*N* = *90*	
Always	138 (78.4)	58 (67.4)	80 (88.9)	
Usually	33 (18.8)	27 (24.9)	9 (10.0)	
Sometimes	4 (2.3)	4 (4.7)	0 (0.0)	
Never	1 (0.6)	0 (0.0)	1 (1.1)	
6 months PP	*N* = *178*	*N* = *87*	*N* = *91*	
Always	124 (69.7)	37 (42.5)	87 (95.6)	
Usually	52 (29.2)	49 (56.3)	3 (3.3)	
Sometimes	2 (1.1)	1 (1.1)	1 (1.1)	
Never	0 (0.0)	0 (0.0)	0 (0.0)	

^a^Interaction effect of study group and time of assessment, no statistically significant difference at baseline, MIHOW < MEI 2 weeks, 2- and 6-months: p < 0.001

^b^Main effect of study group (MIHOW < MEI)

^c^Main effect of study group (MIHOW > MEI)

#### Outcomes in the Linkages and Referrals Domain

As shown in Table 6, there were statistically significant differences between the groups in the receipt of and follow through with referrals. Women in the MIHOW group received more referrals for additional services from community providers than did those in the MEI group (80–100 vs. 22–28%, d = 1.77, p < 0.001), connected with those resources (65–81 vs. 56–54%, d = 0.31, p = 0.028), and received more new services (64–80 vs. 56–54%, d = 0.30, p = 0.035) than women in the MEI group.

**Table 6:** Summaries of linkages and referrals by study group (N = 178)

	Overall	MEI	MIHOW	*p*-value
	N, n (%)			
Referred to resources^a^				< 0.001 (*d* = 1.77)
Prenatal	172, 89 (51.7)	83, 18 (21.7)	89, 71 (79.8)	
6 months PP	178, 115 (64.6)	87, 24 (27.6)	91, 91 (100.0)	
Of referrals, made appointments/visits				0.028 (*d* = 0.31)
Prenatal	89, 56 (62.9)	18, 10 (55.6)	70, 46 (64.8)	
6 months PP	114, 86 (75.4)	24, 13 (54.2)	90, 73 (81.1)	
Of referrals, received new services^a^				0.035 (*d* = 0.30)
Prenatal	88, 55 (62.5)	18, 10 (55.6)	70, 45 (64.3)	
6 months PP	115, 86 (74.8)	24, 13 (54.2)	91, 73 (80.2)	

^a^None of the reported resources or service types included mental health services

#### Outcomes in the Parenting Practices Domain

Measures and indicators of several types of parenting practices are summarized in Table 7. At all postpartum time points, statistically significant differences between groups for the HOME-IT Inventory scores emerged. Compared to observations of home environments of infants in the MEI group, infants in the MIHOW group experienced a statistically significantly higher level of quality and quantity of stimulation and support at each time of assessment (d = 1.99–2.32, p < 0.001). Furthermore, women in the MIHOW group reported greater frequency of singing songs or telling stories to their child (d = 0.86, p < 0.001). Women in the MIHOW group reported greater frequency of reading to their child (d = 1.53, p < 0.001). While more than 90% of the women in the MIHOW group reported reading three or more times per week to their child at both 2 and 6 months postpartum, fewer than 40% of the mothers in the MEI group reported doing so at either time of assessment (p < 0.001). Rates decreased from 37 to 26% between 2 and 6 months postpartum in the MEI group while the rate increased from 92 to 97% during that period in the MIHOW group (p < 0.039).

**Table 7:** Summaries of parenting practices by study group (N = 178)

	Overall N = 178	MEI N = 87	MIHOW N = 91	*p*-value
	Median [IQR] (min, max) N			
HOME score^b^				
2 weeks PP (max = 26)	19.0 [15–22] (6,25) *177*	15.0 [13–18] (6, 23) 86	21.0 [19–23] (15, 25) 91	< 0.001
2 months PP (max = 32)	24.0 [19–27] (7, 30) 175	19.0 [17–23] (7, 27) 85	27.0 [24–28] (15, 30) 90	< 0.001
6 months PP (max = 45)	37.0 [33–40] (23, 44) 178	33.0 [30–36] (23, 41) 87	40.0 [38–42] (33, 44) 91	< 0.001
2 months PP assessment				
Songs and stories (# Days past week)	5.0 [5–6] (2, 7) 176	5.0 [4–5] (2, 7) 86	6.0 [5–7] (3, 7) 90	< 0.001
Read (# Days past week)	4.0 [0–5] (0, 7) 176	0.0 [0–4] (0, 6) 86	5.0 [4–6] (0, 7) 90	< 0.001

	N (%)			
	Overall N = 176	MEI only N = 86	MIHOW N = 90	
Reads stories ≥ 3 times per week				0.039^a^
2 months PP	115 (65.3)	32 (37.2)	83 (92.2)	
6 months PP	109 (61.9)	22 (25.6)	87 (96.7)	

^a^Interaction effect of study group and time of assessment. MEI decreased at 6-months compared to rate at 2-months; MIHOW group remained at similar level; overall main effect of study group: p < 0.001

^b^The Home is essentially a different measure at each time of assessment therefore only group differences at each time of assessment were conducted

## Conclusions for Practice

Using an intent to treat approach, the majority of our hypotheses were supported and provide strong evidence of the effectiveness of MIHOW on improving health outcomes in this sample of Hispanic mothers and their infants. Overall, women assigned to the MIHOW group had fewer depressive symptoms and less parenting stress and more social and emotional help, and better infant feeding and safe sleep practices.

Similar to national statistics (U.S. Department of Health and Human Services 2013), the majority of women in our study entered prenatal care early, underscoring the opportunity to engage with Hispanic families during this time period. As noted in our findings, the women assigned to MIHOW had many better outcomes than women who only received the MEI.

Coupling a home visitation program by trained peer mentors such as MIHOW with standard prenatal care has potential to improve maternal and child health outcomes. At all postpartum time points, women in the MIHOW group reported fewer depressive symptoms, less parenting stress and more social and emotional help than women in the MEI group. These findings depict a mother who may be more able to effectively engage with her infant (Gress-Smith et al. 2012; Nelson et al. 2016). At all postpartum time points, data collectors (blinded to group assignment and using a standardized tool) observed a higher level of quality and quantity of stimulation and support available to the child in the home environment in mothers assigned to MIHOW than in women assigned to the MEI group. Women in the MIHOW group also reported a greater frequency of singing songs, telling stories and reading books to their child than mothers in the comparison group. All of these activities are precursors to appropriate child development and school readiness (Nelson et al. 2016).

In regards to breastfeeding practices, as in other samples of Hispanic mothers (Flores et al. 2016; U.S. Department of Health and Human Services 2013), most women in the study initiated breastfeeding. We did not find any differences between groups for breastfeeding rates at 6 months postpartum or duration of breastfeeding. Our findings of approximately 84% of all study participants providing some breastfeeding is consistent with national data (U.S. Department of Health and Human Services 2013) and exceeds the related Healthy People 2020 (Office of Disease Prevention and Health Promotion 2017) objective (Office of Disease Prevention and Health Promotion 2017). Although not yet attaining the HP2020 objective related to exclusive breastfeeding at 6 months (i.e., 25.5%), women in the MIHOW group did report more breastfeeding exclusivity at 6 months postpartum and longer duration of exclusive breastfeeding. Breastfeeding self-efficacy also was higher in the intervention group at all postpartum time points. Longer duration of breastfeeding exclusivity has many potential benefits for both the mother and the infant (Victora et al. 2016).

Infant mortality rates are generally low among Hispanic families (U.S. Department of Health and Human Services 2013) Safe sleep practices are critical to reducing infant mortality, particularly sudden infant death syndrome (SIDS) (American Academy of Pediatrics 2000). While both groups of mothers met or exceeded a reported national rate (i.e., 65%) for placing their infants on their back to sleep, almost 100% of MIHOW mothers reported at all time points placing their infants on their backs. MIHOW families reported much less co-sleeping than families in the MEI group.

Identifying maternal and family needs and providing appropriate referrals are important aspects of maternal child health care. Follow through on referrals is often low in all racial and ethnic groups (Anisfeld et al. 2004). In this study, both receipt of and follow through with referrals was greater in the MIHOW group than in the MEI group. Shared language and cultural background between study participants and peer mentors may have enhanced participant motivation and ability to follow through with referrals to new services (Andrews et al. 2004).

### Study Limitations

Our study has both strengths and limitations. The use of a randomized controlled design minimized potential for bias. This study had a very high retention rate of participants as compared to other home visitation studies with Hispanic participants (Nguyen et al. 2003). MIHOW extensively trains women from the community being served to conduct the home visits. We also used data collectors who spoke Spanish and were from the same community. We believe the match of peer mentors and data collectors with the study participants added to the successful retention of participants. One notable limitation of the study design was that because all participants received a standard packet of printed educational materials, we did not have a true control group. While this may have made finding differences between groups more difficult, most differences between our groups yielded large effect sizes. The length of the study is another limitation. While the MIHOW intervention is designed to continue until children reach 3 years of age, the duration of the study’s funding only allowed outcomes to be measured until 6 months postpartum.

## Conclusion

Findings from this study expand the limited empiric evidence related to home visitation services. It demonstrates beneficial effects of a well-trained and supervised peer-to-peer model for a sample of Hispanic mothers and infants. Understanding the nuances of providing services to both majority and minority families in the context of a changing medical care landscape is necessary to providing quality care and developing appropriate policy. Results provide strong support of the efficacy of the MIHOW program and the potentially high retention rates for participants. This program should be considered when planning home visitation services for childbearing immigrant and underserved families. MIHOW has established standards of practice, a research-based curriculum, and an accreditation component, along with decades of experience in several states. It is also cost effective. Even with the extensive training, intensive support, and supervision needed for peer mentors, program costs are usually less, overall, than models requiring early childhood or medical professionals to conduct home visits. Additional longitudinal studies are needed to further understand the sustained impact of MIHOW on health outcomes.
