# Correction to: The Efficacy of Using Peer Mentors to Improve Maternal and Infant Health Outcomes in Hispanic Families: Findings from a Randomized Clinical Trial

**DOI:** 10.1007/s10995-018-2625-8

**Published:** 2018-08-22

**Authors:** Melanie Lutenbacher, Tonya Elkins, Mary S. Dietrich, Anais Riggs

**Affiliations:** 1 Schools of Nursing and Medicine (Pediatrics), Vanderbilt University, Nashville, TN USA; 2 Maternal Infant Health Outreach Worker Program, Vanderbilt University, Nashville, TN USA; 3 Schools of Medicine (Biostatistics, Psychiatry, VICC) and Nursing, Vanderbilt University, Nashville, TN USA; 4 Catholic Charities of Tennessee, Inc., Nashville, TN USA

## Correction to: Maternal and Child Health Journal 10.1007/s10995-018-2532-z

The article “The Efficacy of Using Peer Mentors to Improve Maternal and Infant Health Outcomes in Hispanic Families: Findings from a Randomized Clinical Trial”, written by Melanie Lutenbacher, Tonya Elkins, Mary S. Dietrich and Anais Riggs, was originally published electronically on the publisher’s internet portal (currently SpringerLink) on 31 May 2018 without open access. With the author(s)’ decision to opt for Open Choice the copyright of the article changed on 16 July 2018 to © The Author(s) 2018 and the article is forthwith distributed under the terms of the Creative Commons Attribution 4.0 International License (http://creativecommons.org/licenses/by/4.0/), which permits use, duplication, adaptation, distribution and reproduction in any medium or format, as long as you give appropriate credit to the original author(s) and the source, provide a link to the Creative Commons license and indicate if changes were made.

The original article has been corrected.

